# Structure and release properties of pyrethroid/sulfobutyl ether β-cyclodextrin intercalated into layered double hydroxide and layered hydroxide salt

**DOI:** 10.3389/fchem.2022.894386

**Published:** 2022-08-05

**Authors:** Xiaoguang Zhang, Jiexiang Liu, Jihui Ren

**Affiliations:** ^1^ College of Chemistry, Nankai University, Tianjin, China; ^2^ School of Chemical Engineering, Hebei University of Technology, Tianjin, China

**Keywords:** LDH, hybrids, pyrethroid, SBECD, release behavior, LHS

## Abstract

The aim of this study was to realize the intercalation of the pyrethroid pesticides beta-cypermethrin (BCT) and lambda-cyhalothrin (LCT) into ZnAl-layered double hydroxides (LDH) and NiZn-layered hydroxide salt (LHS). BCT (LCT)/SBECD-LDH and BCT (LCT)/SBECD-LHS hybrids were obtained with the aid of sulfobutyl ether β-cyclodextrin (SBECD) through one step method. The hybrids were characterized by powder X-ray diffraction (XRD), Fourier transform infrared spectroscopy (FT-IR), and thermogravimetry and differential thermal analysis (TGA/DTA). The hybrids based on LHS had larger basal spacing than those on LDH. The LDH-hybrids prepared in N-methylpyrrolidone (NMP) had larger basal spacing than those in ethanol. These results were discussed in terms of the matrix structure and solvent properties. The supramolecular structure of the hybrid was reasonably proposed. Furthermore, the release properties of BCT (LCT) from the hybrids were investigated and discussed in two media. The release rate in pH = 5.0 was slower than that in pH = 6.8. The accumulated release amount of pesticide in pH = 5.0 was lower than that in pH = 6.8. LHS-hybrids synthesized in ethanol exhibit a sustainable release property. These depend on the inclusion complexes’ arrangement and release medium. The release kinetic processes could be described by pseudo-second order and parabolic diffusion models. The release behavior can be controlled by adjusting the synthesis conditions and the releasing media. This provides the guidance for the application of SBECD and LDH (LHS) in pesticide formulation.

## Introduction

Layered double hydroxides (LDH) and layered hydroxide salts (LHS) are two kinds of layered material. The structures of LDH and LHS are modifications of the brucite (Mg(OH)_2_)-like structure. The brucite structure can undergo compositional changes when a trivalent cation of M^3+^ isomorphically replaces a part of Mg^2+^, forming an excess of charge in the layers that must be neutralized by interlayer anions. These modifications cause the formation of a series of compounds called LDH (Cavani et al., 1991; Evans and Slade, 2006). LDH have been extensively studied as catalysts (Cavani et al., 1991; Liu et al., 2018; Xu et al., 2018; Laipan et al., 2020a; Yue et al., 2020), adsorbents (Jawad et al., 2017; Zhou et al., 2018; Laipan et al., 2020b), controlled release supports for drugs (Dong et al., 2010; Rives et al., 2013; Rives et al., 2014), and pesticides (Wang et al., 2005; Quan et al., 2009). Brucite-like structures can experience other modifications, but instead of metal replacement, hydroxide ions are removed from the structure and replaced by water molecules or other types of oxoanions in order to balance the charge of the layers. These modifications lead to a type of compounds called LHS. LHS has similar anion exchange property to LDH (Arizaga et al., 2007; Rojas and Giacomelli, 2013). LHS has been widely investigated in various application fields (Yang et al., 2007; Costantino et al., 2008; Cursino et al., 2010; Marangoni et al., 2010; Demel et al., 2011; Biswick et al., 2012; Liu et al., 2015b; Rojas et al., 2015). The zinc hydroxide nitrate (ZHN) with Zn^2+^ in the octahedral sites of the layer has been the most studied subject as an intercalated matrix. The drugs (ibuprofen, naproxen, and ketoprofen) have been intercalated into ZHN through coordinate bond (Rojas et al., 2015). ZHN hybrids have larger basal spacing than LDH. In addition, the thermal stability of NiZn-LHS is much higher than that of ZHN, and NiZn-LHS can be easily synthesized in the range of pH = 6.5–9.0 (Liu et al., 2015a; Liu et al., 2015c). Therefore, ZnAl-LDH and NiZn-LHS are investigated and compared as the intercalated matrix in this work.

As a class of macrocyclic compounds, cyclodextrins (CDs) and cucurbituril (CB [*n*]) have been widely applied in the pharmacy, agricultural, food, and cosmetic industries (Zhu et al., 2007; Zhou et al., 2008; Çelik et al., 2011; Sun et al., 2015; Chen et al., 2018; da Silva et al., 2011; Szejtli, 1998; Zhang et al., 2022). As mentioned earlier, LDH and LHS materials have been used as carriers for various compounds. How about the effect of both LDH (LHS) layers and macrocyclic compounds together on the properties of guest compounds? Neutral CB [6, 7] can be intercalated into the galleries of LDH by using the double-phase anion exchange method (da Silva et al., 2011). The intercalation is due to hydrogen bonding interactions between the carbonyl groups and OH groups of the LDH host layers. And also, the insertion of Ca/β-CD complex into LDH did not destroy the crystal structure of LDH. Ca/β-CD-LDH can convert glycerol into glycerol carbonate (GC) by transesterification of dimethyl carbonate (DMC) and glycerol (Liu et al., 2021). And the glycerol conversion and the GC yield were 93.7% and 91.8%, respectively, when the molar ratio of DMC and glycerol was 3:1. Ca/β-CD-LDH greatly improves the glycerol conversion compared to LDH. The combination of LDH layers and the cavity of carboxymethyl β-cyclodextrin (CMCD) imposes strong restrictions on the mobility of naphthalene and dodecylbenzene molecules in the interlayer space of CMCD-LDH (Mohanambe and Vasudevan, 2005; Liu et al., 2008). In addition, CMCD-LDH could include fragrance and prolong the fragrance release time in comparison to that of LDH (Ciobanu et al., 2013). Sulfonated β-cyclodextrin (SCD) intercalated into LDH has exhibited high selective adsorption for phenolic compounds (Xue et al., 2014). The magnetic adsorbent iron oxide-LDH modified with dodecylsulfate (DS) and β-CD (Fe/LDH-DS/β-CD) can adsorb phenol (PHE), p-nitrophenol (PNP) and p-cresol (PCS) from aqueous solution through a magnetic field (Balbino et al., 2020). The maximum adsorption amounts of PHE, PNP, and PCS by the adsorbent are 216.08, 255.63, and 272.48 mg g^−1^, respectively. The adsorption is spontaneous and endothermic. And the ferrocene molecules were inserted into the interlayer space of SCD-LDH, which efficiently improves the smoke suppression of epoxy resin (Li et al., 2019). Additionally, the drug prazosin has been successfully intercalated into the interlayer space of SBECD-LDH, and the hybrid displays controlled release behavior (Nakayama et al., 2008). The inclusion complexes of the drug 5-fluorouracil/CMCD have been intercalated into the galleries of LDH (Jin et al., 2010). The release rate of 5-fluorouracil was smaller in pH = 4.8 than in pH = 7.2, and the release amount was more in pH = 7.2. Compared to pure LDH, the inclusion complexes inserted into the interlayer space of LDH can significantly improve the properties of guest molecules. How about pyrethroid release from pyrethroid/SBECD-LDH (LHS) hybrids? What is the effect of LDH and LHS on pesticide release? This needs further investigation in the following.

Solvents are indispensable during the hybrids’ preparation. Many investigations have shown that the solvent has important influence on the structures and properties of the composites. Demel et al. (2014) have shown that the basal spacing shrank after sodium dodecyl sulfate-ZHN was further treated with solvents. And also, decarbonation behavior of MgAl-LDH in alcohols (methanol and ethanol) depended on the pH values of ammonium hydrochloride salts (Iyi and Yamada, 2012). The alcohol acts as a proton source in the exchange reaction. In addition, the acid resistance of LDH is much higher in alcohols (methanol and ethanol) than in water, and complete decarbonation has been attained, yielding LDHs that contain the conjugated base anions of the acids (Iyi et al., 2011). Furthermore, the CO_3_^2−^ of MgAl(NiAl)-LDH can be quantitatively replaced by a variety of monovalent anions (chloride, bromide, iodide, nitrate, and acetate) and a divalent sulfate anion in butanol (Bhojaraj et al., 2019). The products retain the crystallinity and morphology of the precursor LDH in most cases. For the inclusion complexes, guest molecules are usually included in the cavity of CDs with the aid of polar solvents. Polar solvents may provide a medium with favourable condition for the exchange reaction of composites. Thus, ethanol and NMP solvents have been selected for the synthesis of hybrids based on LDH (LHS).

Two kinds of important insecticides in the pyrethroid family, beta-cypermethrin (BCT) and lambda-cyhalothrin (LCT), are used widely on cotton, vegetables, and fruit trees, etc. In this work, BCT (LCT) are separately intercalated into the galleries of SBECD-LDH and SBECD-LHS by partitioning the solvents of ethanol (NMP) and water, respectively. The effect of solvents and carrier types on the structure and properties of the hybrids are systematically investigated by powder X-ray diffraction (XRD), Fourier transform infrared spectra (FT-IR), and thermogravimetric analysis and differential thermal analysis (TGA/DTA) technologies. Additionally, the release properties of BCT (LCT)/SBECD-LDH and BCT (LCT)/SBECD-LHS are studied and compared in two buffer solutions (pH = 5.0 and 6.8), and the pseudo second-order and parabolic diffusion models are used to fit the release data. The release mechanism is discussed from the point of the release media and the arrangement models. This study intends to provide support for the potential application of these composites in the field of pesticide formulation.

## Experimental

### Materials

Sulfobutyl ether β-cyclodextrin (SBECD) was obtained from Shandong Binzhou Zhiyuan Biotechnology Co., Ltd.. The degree of substitution (DS) is 6.2–7.0. The pesticides beta-cypermethrin (94.6wt%) and lambda-cyhalothrin (96.2wt%) were obtained from Jiangsu Changlong Chemical Co., Ltd. Their structures are shown in Supplementary Figure S1. N-methylpyrrolidone (NMP) (analytical reagent) was bought from Tianjin Bodi Chemical Co., Ltd., China. Sodium hydroxide, nitric acid, and ethanol absolute were analytical grade and obtained from North Tianjin Pharmacy Chemical Reagent, China; Zn(NO_3_)_2_·6H_2_O, Al(NO_3_)_3_·9H_2_O, and Ni(NO_3_)_2_·6H_2_O were analytical grade and purchased from Beijing Innochem Scientific Ltd., China. Double distilled water was used during the experiments.

### Synthesis of the composites

The precursor ZnAl-LDH (Zn/Al molar ratio = 1.5) was prepared by the coprecipitation method from a mixed solution of zinc and aluminum nitrate (Xie, 2006), and NiZn-LHS (Ni/Zn molar ratio = 1) was from a mixed solution of zinc nitrate and nickel nitrate. The procedure was described as follows: Mixed salt solutions of 1.0 mol L^−1^ Zn (NO_3_)_2_ and 0.5 mol L^−1^ Al(NO_3_)_3_ [or 1.0 mol·L^−1^Ni(NO_3_)_2_] were prepared. Then 0.5 mol L^−1^ sodium hydroxide solution was added dropwise to the mixed solutions until pH = 6.5 under vigorous stirring and a N_2_ atmosphere. The slurry was continuously stirred for 0.5 h and then transferred into a teflon-lined stainless-steel autoclave and kept at 100°C for 3 h. After cooling to an ambient temperature, the slurry was centrifuged, followed by washing three times with double distilled water. The final sediment was dried in an oven at 85°C for 12 h to obtain pristine ZnAl-LDH and NiZn-LHS samples.

**FIGURE 1:**
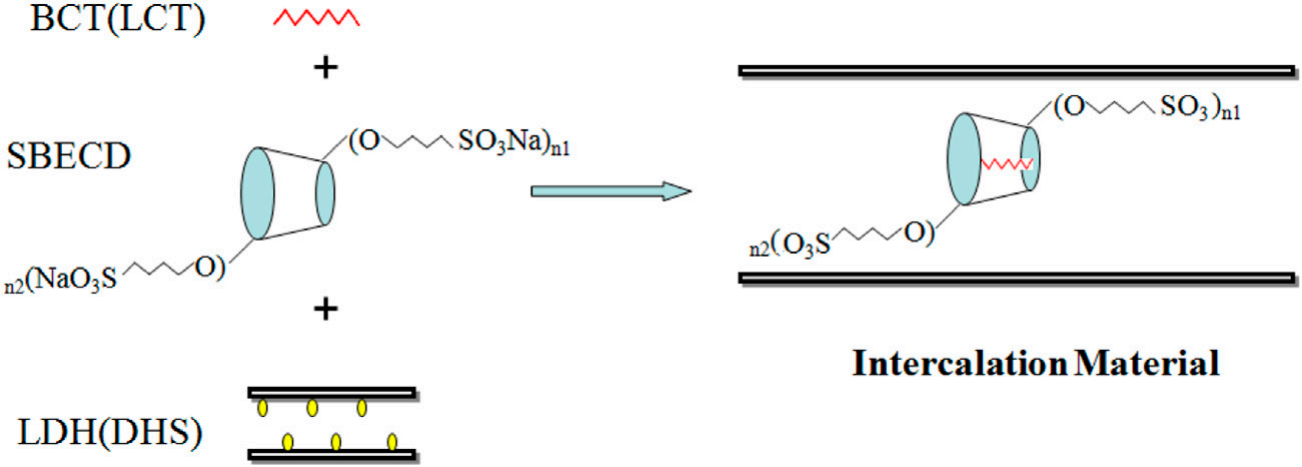
Preparation schematics of the hybrids (SBECD, *n*1+*n*2 = 6.5).

BCT (LCT)/SBECD-LDH and BCT (LCT)/SBECD-LHS hybrids were prepared by a one-pot method, and the preparation schematics are displayed in Figure 1. In this method, a transparent mixture of two solutions is prepared, one consists of 8 ml 0.0347 mol L^−1^ SBECD water solution and 30 ml 0.048 (0.0444) mol L^−1^ BCT (LCT) absolute alcohol solution; the other consisted two solutions of 9 ml 0.0308 mol L^−1^ SBECD water solution and 24 ml 0.06 (0.0556) mol L^−1^ BCT (LCT) in NMP solution. A sample of 0.6 g ZnAl-LDH (NiZn-LHS) was added to the solution above. The pH value was maintained at 6.5, and the mixed dispersion was ultrasonicated for 0.5 h, and stirred under N_2_ atmosphere at 50°C for 48 h. The precipitate was washed extensively with ethanol and water, respectively, centrifuged and dried at 80°C for 12 h. The samples were denoted as BCT (LCT)/SBECD-LDH and BCT (LCT)/SBECD-LHS, respectively.

### Characterization

The crystal structures of the hybrids were characterized by a D8 Focus X-ray diffractometer (Bruker, Germany) equipped with Ni filtered Cu Kα radiation. Fourier-transform infrared spectra were performed on a Tensorn 27 (Bruker, Germany) in air at room temperature by the KBr disc technique. Thermogravimetric analysis and differential thermal analysis were recorded on a SDT Q600 synchronization thermoanalyzer (TA company, United States) to evaluate the thermal stability of the composites. The ultraviolet–visible (UV-Vis) spectra were measured on a TU1901 spectrophotometer (Beijing Purkinje General Instrument Company, China) to measure the pesticide amount of the samples.

### Release studies

#### Determination of beta-cypermethrin (lambda-cyhalothrin) loading

The amount of pesticides loaded in the composites was determined by UV–Vis spectroscopy. A known amount of the sample power was placed in a 25 ml volumetric flask. An appropriate amount of 1 mol L^−1^ HCl **(**about 2–3 ml**)** was added to dissolve the layer, and the remaining space of the flask was filled with ethanol. The suspension was ultrasonicated for 0.5 h, and then was filtered with a Millipore 0.45 μm membrane filter. Then the pesticide concentration in the solution was determined through the absorbance value at 277 nm, and the concentration was calculated by regression analysis based on the standard curve of the pesticide solution. The final value was an average of the measurement results of three parallel samples.

#### Determination of release behavior

A known amount of sample power was dispersed in 200 ml buffer solution at pH = 5.0 (6.8) under magnetic stirring at 30°C. The buffer solution at pH = 5.0 (6.8) was achieved by the phosphate solutions. Aliquots (4 ml) of supernatant were taken at predetermined time intervals, and at the same time 4 ml fresh medium were added in the system. The withdrawn supernatant was immediately filtered through a 0.45 μm syringe filter, and their BCT (LCT) contents were determined by examining the absorbance at 277 nm to obtain the pesticide release amounts and then calculating the accumulated release amounts from the samples. Release tests were conducted repeatedly for three times, and the final results were recorded as an average.

#### Release kinetics

In order to deeply understand the release mechanism, the release data were further fitted with 1) pseudo second-order, 2) parabolic diffusion, and 3) Bhaskar models:

$$\frac{t}{X_{t}}=\frac{t}{X_{e}}+\frac{1}{k\;X_{e}^{2}}$$ (1)

$$\frac{X_{\text{t}}}{t}=k_{d}t^{-0.5}+m$$ (2)

$${-\text{lg }\left(1-\text{Xt}\right)\;=\text{ k}}_{\text{B}}{\text{ t }}^{\text{0}\text{.}65}\;$$ (3)

In above equation, where *X*_t_ and *X*_e_ are the release percentage at any time (*t*) and equilibrium time, respectively, *k* (*k*_*d*_ *, k*_B_) is the corresponding rate constant of release kinetics. If the kinetics model is feasible, the plot of $t/X_{t}\;vs.\;t,\;X_{t}/t\;vs.\;t^{-0.5}$ , and lg (1-*X*_t_) vs. *t* ^0.65^ will be linear, and the *k* value can be obtained from the slope of the fitted curve.

## Results and discussion

### Characterization of beta-cypermethrin (lambda-cyhalothrin)/sulfobutyl ether β-cyclodextrin-layered double hydroxides and beta-cypermethrin (lambda-cyhalothrin)/sulfobutyl ether β-cyclodextrin-layered hydroxide salt

#### X-ray diffraction analysis

**FIGURE 2:**
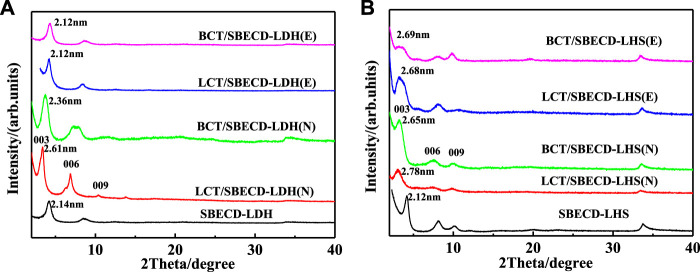
XRD patterns of BCT (LCT)/SBECD-LDH **(A)** and BCT (LCT)/SBECD-LHS samples **(B)** E and N in parentheses denote the solvent ethanol and NMP used in the synthesis of the sample, respectively.

Figure 2 shows XRD patterns of BCT (LCT)/SBECD-LDH and BCT (LCT)/SBECD-LHS samples prepared in the solvents of ethanol and NMP, respectively. The E and N in parentheses denote ethanol and NMP used in the synthesis of the sample, respectively. SBECD-LDH and SBECD-LHS results are also displayed for comparison. In addition, XRD patterns of LDH, LHS, and SBECD are presented in Supplementary Figure S2. The *d* value is the interlayer distance of the layered material. For SBECD-LDH (Figure 2A), the first diffraction peak at 4.20° which corresponds to the basal spacing of 2.14 nm, which is far bigger than that of LDH (2θ = 10.1°, *d* = 0.88 nm) (Jin et al., 2010), suggesting the intercalation of SBECD into the interlayer of LDH. The *d* value of SBECD-LDH is similar to that of SBECD-LDH (2.15 nm) (Nakayama et al., 2008), and is bigger than those of CMCD-LDH (1.52 nm) (Jin et al., 2010), SCD-LDH (1.52 nm) (Xue et al., 2014), and β-CD-LDH (0.76 nm) (Balbino et al., 2020). The size of SBECD is bigger than that of CMCD, SCD, and β-CD, and SBECD may adopt a parallel monolayer in the interlayer space, while CMCD, SCD, and β-CD take a monolayer vertical arrangement (Jin et al., 2010; Xue et al., 2014; Balbino et al., 2020). Hence, SBECD-LDH has a larger interlayer distance than those of CMCD (SCD and β-CD)-LDH. The *d* values of BCT/SBECD-LDH (E) and LCT/SBECD-LDH (E) are 2.12 nm, and are slightly smaller than those of prazosin/SBECD-LDH (2.28–2.35 nm). BCT (LCT) molecules are not soluble in water and are easily included in the cavity of SBECD due to their hydrophobicity. Compared to SBECD-LDH, BCT (LCT) molecules should be located in the cavity of SBECD in the BCT (LCT)/SBECD-LDH (E) hybrids. For BCT/SBECD-LDH (N) and LCT/SBECD-LDH (N) hybrids, the first diffraction peaks are located at 3.72 and 3.36°, corresponding to the *d* values of 2.36 and 2.61 nm, respectively. The *d* values of BCT (LCT)/SBECD-LDH (N) are bigger than those of BCT (LCT)/SBECD-LDH (E). This suggests that some pesticide molecules enter the cavity of SBECD, and others in the interlayer space, which enlarges the interlayer distance. In Figure 2B, the first diffraction peak of SBECD-LHS is similar to that of SBECD-LDH, and the *d* value is 2.12 nm. The first diffraction peaks of LCT/SBECD-LHS (N), BCT/SBECD-LHS (N), LCT/SBECD-LHS (E), and BCT/SBECD-LHS (E) are located at 3.17, 3.30, 3.29, and 3.29°, corresponding to the *d* values of 2.78, 2.65, 2.68, and 2.68 nm, respectively, and are much bigger than that of SBECD-LHS. The *d* of LCT/SBECD-LHS (N) is slightly larger than that of LCT/SBECD-LHS (E). These suggest that BCT (LCT) molecules enter into the galleries of LHS hybrids besides the cavity of SBECD. In addition, the *d* values of BCT (LCT)/SBECD-LHS are bigger than those of BCT (LCT)/SBECD-LDH, which should be attributed to the different structures of LHS and LDH. The zinc tetrahedrons in LHS lead to the increased thickness of composites (Newman and Jones, 1999). The basal spacing of the composites prepared in NMP is bigger than those in ethanol, which is due to the stronger dissolving capacity of NMP for pesticides and the miscibility of NMP with water in the presence of pesticide. The pesticide concentration in NMP is far higher than that in ethanol. This causes more BCT (LCT) molecules to intercalate into the interlayer space, which is in agreement with their loaded amounts in the latter. For the hybrids, β-CD is a neutral cyclodextrin and its intercalation is due to the hydrogen bonding between β-CD and LDH (Balbino et al., 2020). As an anionic cyclodextrin, the intercalation of SBECD is through electrostatic interaction and hydrogen bonding between CD and LDH (LHS). This is also used by Xue et al. (2014) to explain SCD intercalation into LDH. In the following part, FT-IR and TGA/DTA techniques have been used to confirm the existence of BCT (LCT) and SBECD in the composites.

#### Fourier transform infrared spectroscopy analysis

**FIGURE 3:**
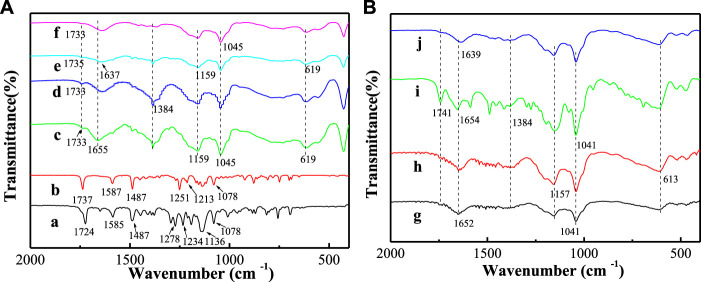
FT-IR spectra of samples **(a)** LCT; **(b)** BCT; **(c)** BCT/SBECD-LDH (N); **(d)** BCT/SBECD-LDH (E); **(e)** LCT/SBECD-LDH (N); **(f)** LCT/SBECD-LDH (E); **(g)** BCT/SBECD-LHS (N); **(h)** BCT/SBECD-LHS (E); **(i)** LCT/SBECD-LHS (N); **(j)** LCT/SBECD-LHS (E).

Figure 3 displays FT-IR spectra of LCT, BCT, LDH-hybrids, and LHS-hybrids. For convenience, FT-IR spectra of LDH, SBECD-LDH, LHS, and SBECD-LHS are presented in Supplementary Figure S3. For LCT, the absorption bands at 3070 and 2970 cm^−1^ are assigned to the C-H stretching vibration of benzene ring and alkyl chain near benzene ring, respectively. The absorption bands at 1585 and 1487 cm^−1^ are attributed to the backbone vibration of benzene ring, respectively. The band at 1724 cm^−1^ comes from the stretching vibration of –COO–. The bands at 1234 and 1278 cm^−1^ are the asymmetric and symmetric stretching vibration of C–O–C, and 1078 cm^−1^ is the asymmetric stretching vibration of phenyl ether. FT-IR spectrum of BCT is similar to that of LCT. The characteristic bands of BCT are located at 1737 and 1078 cm^−1^. These are consistent with dates from the literature (Bi et al., 1995; Zhang et al., 2012). For all samples containing SBECD, the broad bands at 3444 and 1664 cm^−1^ are attributed to the stretching and bending vibration of the OH groups of SBECD, respectively. The band at 2930 cm^−1^ is assigned to the C–H stretching vibration. The bands at 1159, 1045, and 609 cm^−1^ are attributed to the characteristic absorption of glucose units. The adsorption bands at 1250 and 1070 cm^−1^ are attributed to the stretching vibrations of SO_2_ and S-O-C, respectively, indicating the presence of -SO_3_. These assignments are consistent with data from the literature (Xue et al., 2014). For BCT/SBECD-LDH (Figures 3C,D), the broad absorption band at 3480 cm^−1^ is assigned to the stretching vibration of the hydroxyl group in the LDH layers and interlayer water molecules. The band at 1384 cm^−1^ is due to the stretching vibration of interlayer NO_3_^−^ (Jin et al., 2010), which indicates the existence of a little NO_3_^−^ in the sample. The weak adsorption band at 1733 cm^−1^ is attributed to the stretching vibration of –COO– of the BCT molecule. This indicates that BCT exists in the SBECD-LDH hybrid. For LCT/SBECD-LDH (Figures 3E,F), the characteristic band of LCT is observed at 1735 1733) cm^−1^. The characteristic bands of LDH are clearly observed at 3480 and 1384 cm^−1^. This indicates that LCT exists in the interlayer space of the SBECD-LDH sample. For LCT/SBECD-LHS (N) (Figure 3I **)**, the broad band around 3490 cm^−1^ is attributed to the stretching vibration of the hydroxyl group of the layers and interlayer water molecules in DHS. The weak band at 1654 cm^−1^ is assigned to the bending vibration of water molecules. The band at 1384 cm^−1^ belongs to the stretching vibration of interlayer NO_3_^−^ (Liu et al., 2015a). The characteristic band of LCT is apparently observed at 1741 cm^−1^, which suggests that LCT exists in the SBECD-LHS sample. For BCT/SBECD-LHS (N), BCT/SBECD-LHS (E), and LCT/SBECD-LHS (E), the characteristic bands of BCT (LCT) at 1741 cm^−1^ are very weak, which may be correlated to the pesticide content.

On the basis of the abovementioned XRD and FT-IR results, the supramolecular structures of the hybrids can be deduced. The inner diameter, outer diameter, and height of β-CD are 0.60, 1.53, and 0.78 nm, respectively. The length of the sulphobutylether [-OCH_2_(CH_2_)_2_CH_2_SO_3_^−^] group in SBECD molecule is 0.93 nm according to the length of different groups. In addition, the lengths of BCT and LCT molecules are 1.70 and 1.78 nm with the program package DMol^3^ in the Materials Studio of Accelrys, Inc. On the basis of the literature (He et al., 2006; Zhou et al., 2008; Hu et al., 2010), it is deduced that the nonpolar phenyl ether of the BCT (LCT) molecule lies inside the hydrophobic cavity of SBECD and the other is located at the outside of the cavity. The *d* of BCT (LCT)/SBECD-LDH (E) is 2.12 nm. By subtracting the layer thickness of LDH (0.48 nm), the interlayer space available for BCT (LCT)/SBECD is 1.64 nm, which is slightly bigger than the outer diameter of β-CD (1.53 nm), and is much smaller than the sum (0.78 + 0.93 × 2 = 2.64 nm) of the height of β-CD and the length of two sulphobutylether groups. According to the interlayer height and the literature (Wang et al., 2004; Liu et al., 2008; Jin et al., 2010; Xue et al., 2014), the BCT (LCT)/SBECD inclusion anions must adopt a parallel monolayer arrangement in the interlayer space. For BCT/SBECD-LDH (N), the interlayer height is 1.88 nm, which is bigger than 1.53 and smaller than 2.64 nm. Hence, BCT/SBECD inclusion anions also adopt a parallel monolayer in the interlayer space. In the case of LCT/SBECD-LDH (N), the interlayer height is 2.13 nm, which is equal to the sum of the inner diameter (0.60 nm) of β-CD and the outer diameter (1.53 nm), and is smaller than 2.64 nm. Thus the inclusion anions may adopt a parallel monolayer or bilayer arrangement.

The structures of LHS-hybrids are different from those based on LDH. The interlayer heights of LCT/SBECD-LHS (N), BCT/SBECD-LHS (N), LCT/SBECD-LHS (E) and BCT/SBECD-LHS (E) are 1.78, 1.65, 1.68, and 1.69 nm according to the layer thickness of DHS (0.48 nm) and 2 zinc tetrahedrons (0.52 nm), respectively. The interlayer heights of four composites are slightly bigger than the outer diameter of β-CD (1.53 nm) and smaller than the length of SBECD (2.64 nm). Thus, the inclusion anions must be disposed in a parallel single layer arrangement. In addition, the release behavior of the composites may provide a judgment evidence for their supramolecular structure.

#### Thermal gravimetry and differential thermal analysis

**FIGURE 4:**
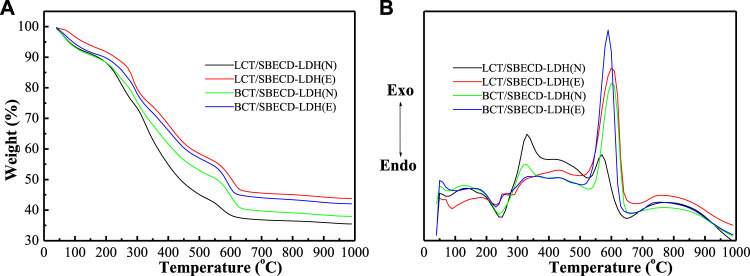
TGA **(A)** and DTA **(B)** curves of BCT (LCT)/SBECD-LDH samples.

Figure 4 shows TGA and DTA curves of LDH-hybrids and LHS-hybrids, respectively. For comparison, TGA and DTA curves of LDH, LHS, BCT, LCT, and SBECD are presented in Supplementary Figure S4. The LDH exhibits two weight loss stages. The first stage (30–130°C) is attributed to the loss of adsorbed water (both the external surfaces and the part gallery), with a corresponding endothermic peak (120°C) in the DTA curve; the second (150–280°C) corresponds to the removal of gallery water, the dehydroxylation of the brucite-like layers, and decomposition of the NO_3_^−^ anions, with a strong endothermic peak at 270°C. For LHS, the adsorbed and gallery water are removed gradually in the range of 30–300°C, and then the TG curve sharply decreases in the range of 300–350°C, which is attributed to the removal of the dehydroxylation of the brucite-like layers and decomposition of the NO_3_^−^ anions. The DTA curve shows a strong endothermic peak at 326°C. For SBECD, the first step (30–100°C) is attributed to the loss of adsorbed and cavity water, accompanied by an endothermic peak at 70°C in the DTA curve; the second step (200–270°C) is due to the fusion and partial decomposition of SBECD, accompanied by a weak endothermic peak at 216°C. The third step (350–690°C) has three exothermic peaks at 360, 623, and 663°C, which can be attributed to the decomposition and complete combustion of SBECD, respectively. The pesticide BCT exhibits three weight loss stages (30–150, 150–350, and 350–600°C), which are melting, decomposition, and combustion stages, corresponding to an endothermic peak (67°C) and two exothermic peaks (331,521°C). The weight loss process of LCT is similar to that of BCT, there are two endothermic peaks (53,310°C) and two exothermic peaks (330,491°C), and the endothermic peak at 310°C is very weak. The endothermic peaks at 67, 53°C are the melting point of BCT, LCT, respectively. In Figure 4, for BCT/SBECD-LDH (N), the first event (40–100°C) is attributed to the loss of adsorbed water and the melting of BCT, accompanied by a weak endothermic peak at 75°C in the DTA curve. The second event (100–300°C) is the fusion and decomposition of SBECD and the dehydroxylation of the brucite-like layer, accompanied by a strong endothermic peak (240°C). The third (300–500°C) is the result of the decomposition and combustion of BCT and SBECD with a sharp exothermic peak (324°C) and a broad exothermic peak (430°C). The exothermic peak (324°C) should be attributed to the decomposition of BCT. The fourth (500–650°C) is attributed to the decomposition and complete combustion of SBECD with a strong exothermic peak (600°C). The thermal behavior of BCT/SBECD-LDH (E) is similar to that of BCT/SBECD-LDH (N). The four weight loss steps of BCT/SBECD-LDH (E) are 40–150, 150–300, 300–500, and 500–650°C, accompanied by two weak endothermic peaks (100 and 230°C) and two exothermic peaks (435 and 590°C). Compared to the first endothermic peak (75°C) of BCT/SBECD-LDH (N), that of BCT/SBECD-LDH (E) increases by 25°C. In addition, the sharp exothermic peak (324°C) is not found. LCT/SBECD-LDH (N) has similar thermal decomposition process to that of BCT/SBECD-LDH (N). LCT/SBECD-LDH (N) has two endothermic peaks (75 and 245°C) and three exothermic peaks (135, 330, and 570°C). LCT/SBECD-LDH (E) has three endothermic peaks (89, 229, and 286°C) and two exothermic peaks (430 and 600°C). Compared to pure SBECD, the combustion temperatures of SBECD in BCT (LCT)/SBECD-LDH decreases apparently. A similar phenomenon has been observed by us Liu et al. (2014). The reason is not clear until now. In addition, compared with the melting point of pure BCT (67°C) and LCT (53°C), those of BCT and LCT in samples increased by 8–33 and 22–36°C, respectively, which indicates that pesticide molecules do not lie on the external surface of the composites.

**FIGURE 5:**
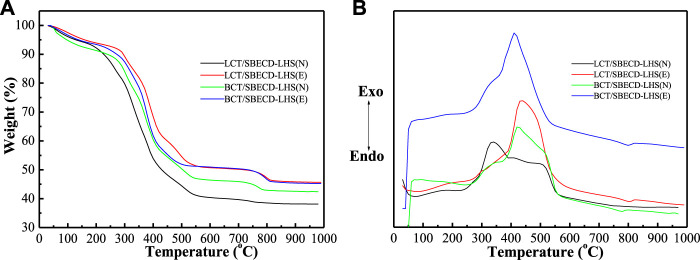
TGA **(A)** and DTA **(B)** curves of BCT (LCT)/SBECD-LHS samples.

In Figure 5, the decomposition process of BCT/SBECD-LHS(N) has three stages, 50–260, 260–380, and 380–600°C in the TGA curve, accompanying a feeble endothermic peak (243°C), a weak exothermic peak (350°C), a strong broad exothermic peak (423°C), and a shoulder peak (513°C) in the DTA curve. The endothermic peak (243°C) should be attributed to the fusion of SBECD. The weak exothermic peak (350°C) may be due to the decomposition of SBECD and BCT. The shoulder peak (513°C) is the combustion of SBECD and BCT. The thermal decomposition behavior of BCT/SBECD-LHS (E) is similar to that of BCT/SBECD-LHS (N). BCT/SBECD-LHS (E) has a weak exothermic peak (340°C) and a strong exothermic peak (411°C) in the DTA curve, and the endothermic peak is not observed. LCT/SBECD-LHS (N) has three stages, 30–250, 250–390, and 390–600°C, accompanying an endothermic peak (70°C), a strong exothermic peak (339°C), and two weak exothermic peaks (400 and 500°C). LCT/SBECD-LHS (E) has an endothermic peak (80°C), a weak exothermic peak (300°C), and a strong and broad exothermic peak (440°C). In addition, LCT/SBECD-LHS(N, E) has endothermic peaks (70, 80°C) which are higher than the melting point (53°C) of pure LCT. These may indicate the different location of pesticide molecules in the hybrids. For LCT/SBECD-LHS, some LCT molecules lie in the cavity of the CD, others lie in the interlayer space, while BCT/SBECD-LHS and BCT molecules mainly lie in the cavity of the CD. Compared to SBECD and BCT (LCT)/SBECD-LDH, the complete combustion temperatures of SBECD in BCT (LCT)/SBECD-LHS decrease up to 70°C. This should be due to the different interaction modes between the LDH (LHS) layers and SBECD. The interaction between LDH layers and SBECD is the electrostatic interaction, while that of LHS and SBECD is the coordinated bond, and the former is stronger than the latter. This has also been used by Rojas et al. (2015)to explain the difference in structure and properties of the hybrids based on LDH and LHS.

### Release of beta-cypermethrin (beta-cypermethrin)/sulfobutyl ether β-cyclodextrin-layered double hydroxides and beta-cypermethrin (beta-cypermethrin)/sulfobutyl ether β-cyclodextrin-layered hydroxide salt hybrids

#### Loading of pesticide

The BCT and LCT loadings of the samples are listed in Table 1. It is found that the solvent has important influence on the loadings of the samples. The samples prepared in NMP have more pesticide loadings than those in ethanol. BCT (LCT)/SBECD-LDH (N) have more pesticide loadings than BCT (LCT)/SBECD-LHS (N). The reason comes from two aspects: one is the strong dissolving capacity of NMP for pesticide, the high miscibility of NMP and water in the presence of pesticide, which leads to higher pesticide concentrations in NMP than ethanol; the other is the difference in interlayer height of the hybrids. The interlayer heights of BCT/SBECD-LDH (N) and LCT/SBECD-LDH (N) are 1.88 and 2.13 nm, which are larger than those of BCT/SBECD-LHS (N) and LCT/SBECD-LHS (N), respectively. The large interlayer space can accommodate more pesticide molecules. In addition, the zinc tetrahedrons of the LHS layer may prevent pesticide molecules from entering the interlayer space and lead to lower loading. The pesticide loading of BCT (LCT)/SBECD-LDH (E) is close to that of BCT (LCT)/SBECD-LHS (E), which is related to their similar interlayer spacing.

**TABLE 1:** Pesticide loading of the samples.

Samples	LCT/SBECD-LDH (E)	LCT/SBECD-LDH (N)	BCT/SBECD-LDH (E)	BCT/SBECD-LDH (N)
The loading (%)	2.1	5.7	1.9	5.3
Samples	LCT/SBECD-LHS(E)	LCT/SBECD-LHS(N)	BCT/SBECD-LHS(E)	BCT/SBECD-LHS(N)
The loading (%)	1.7	3.4	2.0	3.2

Note: E and N denote ethanol and NMP, respectively.

#### Release behavior

**FIGURE 6:**
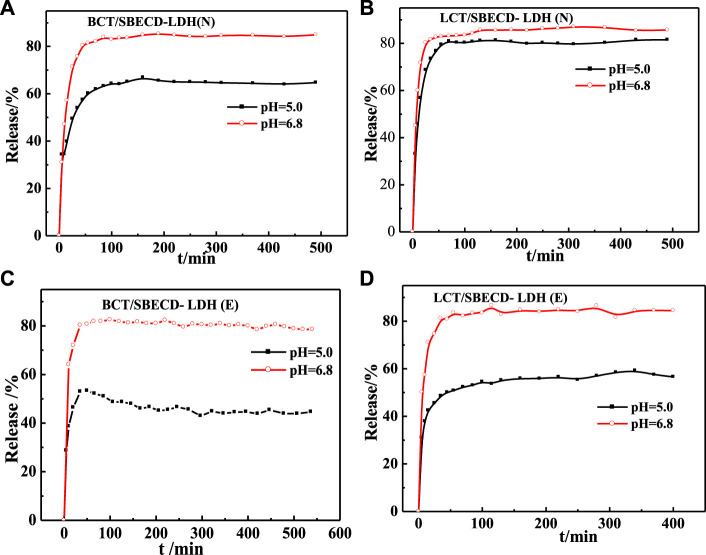
Release curves of pesticides from LDH-hybrids in different media.

Release behaviors of BCT/SBECD-LDH and LCT/SBECD-LDH are shown in Figure 6. For BCT/SBECD-LDH(N), a rapid release of BCT molecules occurs during the initial 35–40 min in two buffer solutions, followed by a slow release until the release equilibrium. The release equilibrium is achieved at about 160 min, and the accumulated released amounts are about 85 and 67% in pH = 6.8 and pH = 5.0, respectively. The release behaviors of LCT/SBECD-LDH (N), BCT/SBECD-LDH (E), and LCT/SBECD-LDH (E) are similar to those of BCT/SBECD-LDH(N). The cumulative released amount at pH = 6.8 is more than 80%, distinctly higher than those in pH = 5.0 (50–80%), which is similar to the release behavior of 5-FU/CMCD-LDH sample (Jin et al., 2010). In addition, the cumulative released amount of BCT (LCT)/SBECD-LDH (E) at pH = 5.0 is far lower than that of BCT (LCT)/SBECD-LDH (N). Moreover, the release properties of BCT (LCT)/SBECD-LDH are completely opposite to those of pesticide intercalated LDH composites directly (Quan et al., 2009; Jia, 2010), in which the lower pH leads to the fast pesticide release from the samples.

**FIGURE 7:**
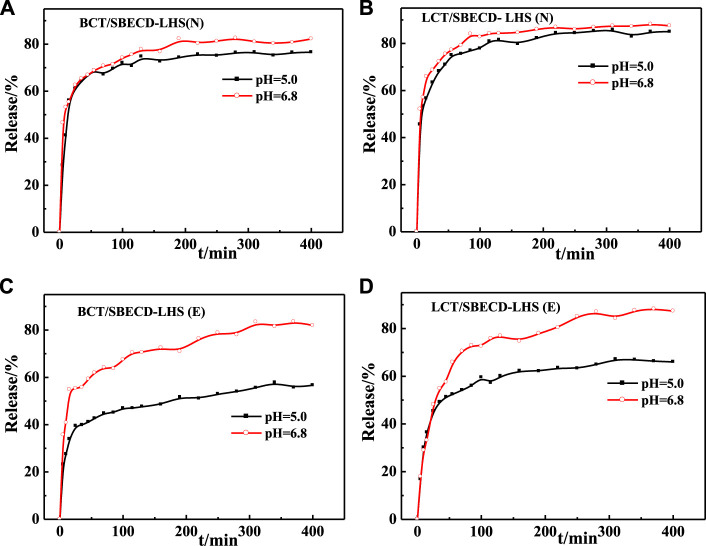
Release curves of pesticides from LHS-hybrids in different media.

Release behaviors of BCT/SBECD-LHS and LCT/SBECD-LDHS are shown in Figure 7. For BCT/SBECD-LHS(N) (Figure 7A), the rapid release of BCT is found during the initial 15 min, followed by a sustained slow release. At pH = 6.8 and pH = 5.0 buffer solutions, the release equilibrium is achieved at about 280 and 310 min, and the accumulated released amounts are about 83 and 77%, respectively. In Figure 7B, the release behavior of LCT/SBECD-LHS (N) is similar to that of BCT/SBECD-LHS (N). At pH = 6.8 and pH = 5.0, the release equilibrium is achieved at about 370 min, and the accumulated released amounts are about 88 and 85%, respectively. For BCT/SBECD-LHS (E) (Figure 7C), the rapid release is found in the initial 20 min, followed by a sustainable slow release. For LCT/SBECD-LHS (E) (Figure 7D), the sustained slow release is observed during the whole process studied, and the released amount at pH = 6.8 is apparently higher than that at pH = 5.0. The released amount of LHS-hybrids at pH = 6.8 is higher than that at pH = 5.0, which is similar to that of LDH.

Comparing Figure 7 with Figure 6, it can be found that the cumulative released amounts of the composites at pH = 6.8 are distinctly higher than those at pH = 5.0, and the release properties of the samples based on LHS are better than those of LDH. The reason for this phenomenon may be that HPO_4_^2−^ at a pH = 6.8 buffer solution has a higher charge density than H_2_PO_4_^−^ at a pH = 5.0, which leads to the higher ion-exchange capacity of HPO_4_^2−^ with the inclusion complexes than that of H_2_PO_4_^−^ “(Duan and Zhang, 2007). Pesticide release from the hybrids could be mainly controlled by the ion-exchange reaction. In addition, the pesticide incorporation into the CD cavities may contribute to the release behavior of the sample (Jin et al., 2010). Furthermore, the release behavior of the pesticide from the hybrid is closely correlated with interlayer space and the arrangement models of the complexes. For BCT (LCT)/SBECD-LDH (E), the interlayer height is 1.64 nm, the inclusion complexes must adopt a parallel monolayer arrangement, and there is suitable space for ion exchange between phosphate anions and inclusion complexes, which benefits BCT (LCT)/CD complexes released rapidly from the sample and pesticide diffusion from the CD cavity to the buffer solution. For BCT/SBECD-LDH (N), the interlayer height is 1.88 nm, which is bigger than that of BCT (LCT)/SBECD-LDH (E). The inclusion anions must adopt a parallel monolayer arrangement. The interlayer space can accommodate more phosphate anions and the inclusion complexes transfer sharply from the sample to the buffer solution, which leads to the high cumulative release amount. Inclusion complexes should adopt a parallel monolayer arrangement from the point of the release behavior. For LCT/SBECD-LDH (N), the interlayer space is 2.13 nm, LCT/CD may adopt a parallel monolayer or bilayer arrangement. As LCT/CD adopts a parallel bilayer arrangement, the LDH layer will delay the inclusion complexes’ release due to the crowded interlayer space. In fact, LCT molecules are released sharply during the initial 50 min, and the release equilibrium is achieved at about 100 min. Furthermore the cumulative released amount of LCT/SBECD-LDH (N) at pH = 5.0 is far higher than that of BCT/SBECD-LDH (N). This suggests that the LCT/CD complexes should adopt a parallel monolayer arrangement, the composite has larger space for anion exchange, and the inclusion anions release rapidly from the sample and diffuse from the cavity to the buffer solution.

The interlayer heights of LCT/SBECD-LHS (ND), LCT/SBECD-LHS (E), BCT/SBECD-LHS (N), and BCT/SBECD-LHS (E) are 1.78, 1.68, 1.65, and 1.69 nm respectively. The inclusion anions adopt a parallel monolayer arrangement according to the sizes of BCT (LCT) and SBECD molecules. In addition, the presence of zinc tetrahedrons in LDHS may lead to crowded space and delay the anion exchange between inclusion anions and phosphate ions, which slows down the pesticide release. Indeed, pesticide release from the composites presents a sustained slow release, especially BCT (LCT)/SBECD-LHS (E).

BCT (LCT) released from the hybrids could be influenced by the following steps: 1) dissolution of the layer; 2) ion exchange between the BCT (LCT)/SBECD inclusion complexes and phosphate anions in buffer solution; 3) the interlayer space of the samples and support structure. The second and third steps may play the dominant role in the release process. The release mechanism of BCT (LCT) from the composite is very complicated and difficult to understand thoroughly. In other words, the release behavior of BCT (LCT) from the sample could be modulated by changing the pH value of the release medium and the solvent used in the preparation process.

#### Release kinetics

**FIGURE 8:**
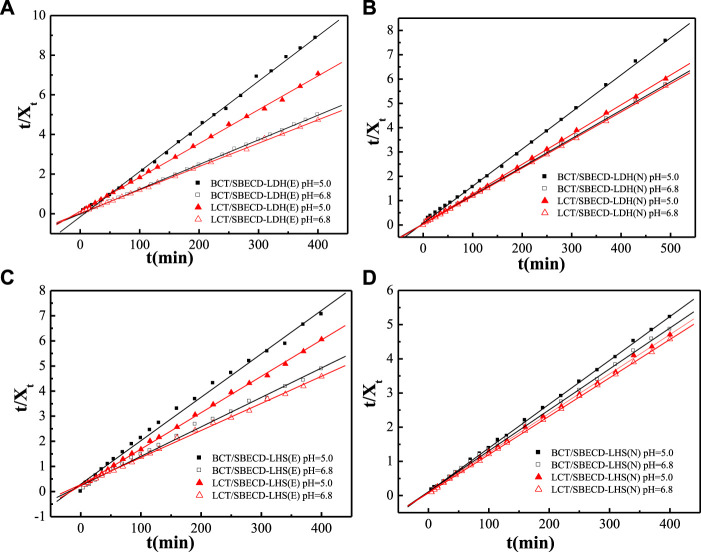
Regression curves of release data fitting with pseudo second-order model for LDH-hybrids **(A,B)** and LHS-hybrids **(C,D)** in different mediums.

**FIGURE 9:**
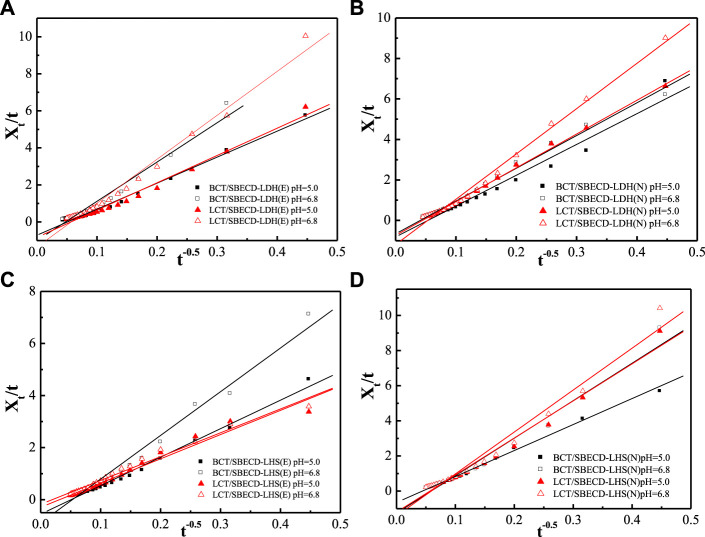
Regression curves of release data fitting with parabolic diffusion model for LDH-hybrids **(A,B)** and LHS-hybrids **(C,D)** in different mediums.

**FIGURE 10:**
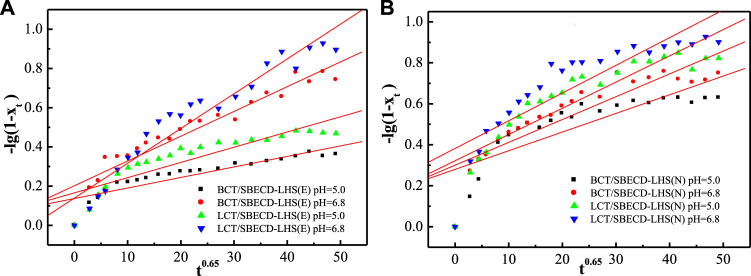
Regression curves of release data fitting with Bhaskar model for DHS-hybrids (E) and LHS-hybrids (N) in different mediums.

To understand the pesticide release mechanisms, the release data are fitted by pseudo second-order kinetic and parabolic diffusion models, shown in Figures 8, 9. The fitted results of rate constants (*k*) and correlation coefficient (*R* ^*2*^) for hybrids are displayed in Table 2. For hybrids of LDH and LHS, pseudo second-order kinetic (*R* ^2^ > 0.9977) and parabolic diffusion models (*R* ^2^ > 0.9826) are more satisfactory for describing the overall release kinetic processes. Researchers have shown that the diffusion-controlled release processes of drug molecules from the clay composites can be described using the parabolic diffusion model (Gu et al., 2008; Wu et al., 2013). The linear fitting results imply that the BCT (LCT) release process is controlled by a diffusion process such as intraparticle diffusion or surface diffusion. In order to clarify the diffusion process, the release data of BCT (LCT)/SBECD-LHS are also simulated by the Bhaskar model, and the results are shown in Figure 10. The rate constants (*k*_B_) and correlation coefficient (*R* ^2^) are displayed in Table 3 (those of BCT (LCT)/SBECD-LDH are not presented as the *R* ^2^ values are lower than 0.8). It has been suggested that the particle diffusion control process could be tested by simply testing for linearity between log (1−X_t_) and t^0.65^. Good linear fittings are found for BCT (LCT)/SBECD-LHS at pH = 5.0 and pH = 6.8 buffer solutions (*R* ^2^ > 0.8212), which suggests that the intraparticle diffusion is the rate-limiting step for these hybrids. This is also used by Zhang et al. (2017a); Zhang et al. (2017b) to explain the drug release from drug-Ch-LDH and (drug-Ch-LDH) @LS hybrids at both pH 7.4 and 4.8 PBS.

**TABLE 2:** Kinetic parameter of different hybrids in pH = 5.0 and = 6.8 mediums.

Samples	Release mediums	Pseudo second-order *k* (10^−3^) *R* ^2^		Parabolic diffusion *k R* ^2^	
BCT/SBECD-LDH (E)	pH = 6.8	4.595	0.9999	21.19	0.9835
	pH = 5.0	3.663	0.9990	14.04	0.9945
LCT/SBECD-LDH (E)	pH = 6.8	5.441	0.9997	23.71	0.9911
	pH = 5.0	2.296	0.9995	14.74	0.9911
BCT/SBECD-LDH (N)	pH = 6.8	2.994	0.9999	16.17	0.9826
	pH = 5.0	3.352	0.9997	15.22	0.9957
LCT/SBECD-LDH (N)	pH = 6.8	4.816	0.9999	22.38	0.9954
	pH = 5.0	3.505	0.9998	16.70	0.9982
BCT/SBECD-LHS (E)	pH = 6.8	0.5919	0.9978	10.94	0.9939
	pH = 5.0	1.007	0.9977	16.79	0.9904
LCT/SBECD-LHS (E)	pH = 6.8	0.4211	0.9991	9.154	0.9904
	pH = 5.0	0.8682	0.9996	9.311	0.9841
BCT/SBECD-LHS (N)	pH = 6.8	1.261	0.9996	21.34	0.9841
	pH = 5.0	1.520	0.9999	14.80	0.9940
LCT/SBECD-LHS (N)	pH = 6.8	1.625	0.9997	23.88	0.9846
	pH = 5.0	1.312	0.9999	21.11	0.9877

**TABLE 3:** Kinetic parameter of Bhaskar model of hybrids in pH = 5.0 and pH = 6.8 solutions.

Samples	Release mediums	*k*_B_	*R* ^2^
BCT/SBECD-LHS (E)	pH = 5.0	0.00537	0.9038
	pH = 6.8	0.0126	0.9547
LCT/SBECD-LHS (E)	pH = 5.0	0.00775	0.8897
	pH = 6.8	0.0177	0.9634
BCT/SBECD-LHS (N)	pH = 5.0	0.00911	0.8212
	pH = 6.8	0.0111	0.8833
LCT/SBECD-LHS (N)	pH = 5.0	0.0128	0.8833
	pH = 6.8	0.0135	0.8663

## Conclusion

The BCT (LCT)/SBECD inclusion complexes have been successfully intercalated into the gallery of LDH (LHS) through partitioning from the solvents of ethanol (NMP) and water. LDH-hybrids prepared in NMP have a larger interlayer height than those in ethanol, which is in agreement with their loaded amount. Though LHS-hybrids prepared in NMP have interlayer height similar to those in ethanol, the loading of the former is far higher than the latter. These are caused by the strong dissolving capacity of NMP and the miscibility of NMP and water in the presence of pesticide. Based on the interlayer height and the sizes of BCT (LCT) and SBECD molecules, the inclusion complexes may adopt a parallel monolayer arrangement in the gallery. The release behaviors of BCT (LCT) are discussed at pH values and arrangement models. DHS-hybrids prepared in ethanol exhibit a better sustainable release than those of LDH. The release behaviors are well described with pseudo second-order and parabolic diffusion models. The present study suggests that polar solvent has an important effect on the structure and properties of the composites. The samples could be potentially applied as a novel controlled-release formulation.

## Data Availability

The original contributions presented in the study are included in the article/Supplementary Material, and further inquiries can be directed to the corresponding authors.
