# Supplementary material for: Structure and release properties of pyrethroid/sulfobutyl ether β-cyclodextrin intercalated into layered double hydroxide and layered hydroxide salt
Source: Front Chem. 2022 Aug 5;10:894386. doi: 10.3389/fchem.2022.894386 (PMC9388771; doi:10.3389/fchem.2022.894386)
Supplement: Supplementary file 1 [file DataSheet1.docx]

**Structure and release properties of pyrethroid/sulfobutyl ether β-cyclodextrin intercalated into layered double hydroxide and layered hydroxide salt**

Xiaoguang Zhang^†,1,^*, Jiexiang Liu^†,2,^* and Jihui Ren^2^

*^1^College of Chemistry, Nankai University, Tianjin 300071, China*

*^2^ School of Chemical Engineering, Hebei University of Technology, Tianjin 300130, China*

**Supporting information**

Structures of beta cypermethrin (BCT) and lambda-cyhalothrin (LCT) (**FIGURE S1**). XRD patterns of the pristine LDH, LHS, SBECD(**FIGURES2**). FT-IR spectra of LDH, SBECD-LDH, LHS and SBECD-LHS (**FIGURE S3**). TGA/DTA curves of LDH, LHS BCT, LCT and SBECD (**FIGURE S4**).

| 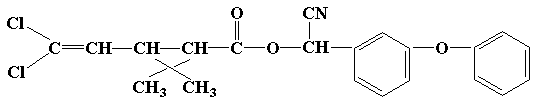 | 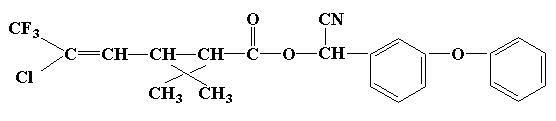 |
| --- | --- |
| BCT | LCT |
| **FIGURES1** Structures of beta cypermethrin (BCT) and lambda-cyhalothrin (LCT) | |

|  |
| --- |
| **FIGURE S2** XRD patterns of LDH, LHS,SBECD, |

|  |
| --- |
| **FIGURE S3** FT-IR of LDH, SBECD-LDH, LHS and SBECD-LHS |

|  |  |
| --- | --- |
| **FIGURE S4** TGA (A) and DTA (B) curves of LDH, LHS, BCT, LCT and SBECD | |
